# Lineage Analysis of the Late Otocyst Stage Mouse Inner Ear by Transuterine Microinjection of A Retroviral Vector Encoding Alkaline Phosphatase and an Oligonucleotide Library

**DOI:** 10.1371/journal.pone.0069314

**Published:** 2013-07-25

**Authors:** Han Jiang, Lingyan Wang, Kevin T. Beier, Constance L. Cepko, Donna M. Fekete, John V. Brigande

**Affiliations:** 1 Department of Otolaryngology, Oregon Hearing Research Center, Oregon Health & Science University, Portland, Oregon, United States of America; 2 Jilin Province Key Laboratory of Animal Embryo Engineering, College of Animal Science and Veterinary Medicine, Jilin University, Changchun, China; 3 Department of Genetics, Howard Hughes Medical Institute, Harvard Medical School, Boston, Massachusetts, United States of America; 4 Department of Biological Sciences, Purdue University, West Lafayette, Indiana, United States of America; University of Washington, Institute for Stem Cells and Regenerative Medicine, United States of America

## Abstract

The mammalian inner ear subserves the special senses of hearing and balance. The auditory and vestibular sensory epithelia consist of mechanically sensitive hair cells and associated supporting cells. Hearing loss and balance dysfunction are most frequently caused by compromise of hair cells and/or their innervating neurons. The development of gene- and cell-based therapeutics will benefit from a thorough understanding of the molecular basis of patterning and cell fate specification in the mammalian inner ear. This includes analyses of cell lineages and cell dispersals across anatomical boundaries (such as sensory versus nonsensory territories). The goal of this study was to conduct retroviral lineage analysis of the embryonic day 11.5(E11.5) mouse otic vesicle. A replication-defective retrovirus encoding human placental alkaline phosphatase (PLAP) and a variable 24-bp oligonucleotide tag was microinjected into the E11.5 mouse otocyst. PLAP-positive cells were microdissected from cryostat sections of the postnatal inner ear and subjected to nested PCR. PLAP-positive cells sharing the same sequence tag were assumed to have arisen from a common progenitor and are clonally related. Thirty five multicellular clones consisting of an average of 3.4 cells per clone were identified in the auditory and vestibular sensory epithelia, ganglia, spiral limbus, and stria vascularis. Vestibular hair cells in the posterior crista were related to one another, their supporting cells, and nonsensory epithelial cells lining the ampulla. In the organ of Corti, outer hair cells were related to a supporting cell type and were tightly clustered. By contrast, spiral ganglion neurons, interdental cells, and Claudius' cells were related to cells of the same type and could be dispersed over hundreds of microns. These data contribute new information about the developmental potential of mammalian otic precursors *in vivo*.

## Introduction

The mouse inner ear and its associated primary sensory neurons develop from a thickened patch of head ectoderm called the otic placode that is established at the start of the second week of gestation. Recent genetic fate mapping data indicate that neuroepithelial cells originating from the neural tube and migrating neural crest cells can also contribute to both the placodal epithelium prior to otic vesicle closure and to the cochleovestibular ganglion [1]. The fully differentiated mammalian inner ear is a structurally complex sensory organ responsible for hearing and balance [2], [3]. The five vestibular sensory organs are the three cristae within the ampullae of the semicircular canals and the two maculae within the saccule and utricle. The cristae and the maculae respond to angular and linear acceleration, respectively. The auditory sensory organ is the organ of Corti within the coiled cochlear duct. The sensory organ types have unique structural and physiological differences but share in common an epithelial layer consisting of mechanically sensitive hair cells and their non-sensory supporting cells [4], [5]. Hearing loss and vestibular pathology are most frequently associated with the death or dysfunction of sensory hair cells or their innervating neurons. The mature mammalian inner ear, in contrast to lower vertebrates, lacks the capacity to regenerate damaged or dead hair cells [6]. There is keen interest in pharmacologic, gene, and cell replacement strategies to restore hearing and balance in the diseased or damaged inner ear [7], [8], [9], [10]. A more complete understanding of how cell fate is specified in the developing mammalian inner ear may inform translational therapies aimed at gene- and cell-based strategies to restore auditory or vestibular function.

Lineage analysis can define the types of cell fate choices made by the progeny of otic epithelial progenitors and the timing of those choices [11]. The association of lineally related cells with anatomical boundaries or known domains of gene expression may provide insight into patterning and cell fate. In the avian inner ear, retrovirus-based lineage analysis has demonstrated that: sensory hair cells and supporting cells can share a common progenitor; vestibular neurons can be clonally related to the mechanosensitive cells they innervate in the paratympanic organ of the middle ear; and that acoustic and vestibular neurons can share a common progenitor [12], [13], [14]. In addition, there is limited dispersion of clonally related cells across anatomical subdivisions in the avian inner ear, suggesting that boundaries of lineage restriction may influence both patterning and cell fate specification [12], [15]. The present study extends lineage analysis of the inner ear to the mouse embryo, which poses the additional technical challenge of *in utero* accessibility, in comparison to the chick embryo growing *in ovo*. Lineage studies were conducted by transuterine microinjection of a replication-defective retroviral vector encoding PLAP as a marker gene and a degenerate oligonucleotide sequence tag as a lineage label [16], [17], [18].

Otic epithelial progenitors in the E11.5 mouse otocyst were targeted for three reasons. First, introduction of the retroviral lineage vector by transuterine microinjection into the otocyst prior to overt differentiation of the sensory epithelia is predicted to label auditory and vestibular sensory precursors. The E11.5 cochlear duct is initially and broadly defined by expression of the Notch ligand, Jagged 1, and the transcription factor, Sox2 [19], [20], [21]. By E12.5, the prosensory domain that gives rise to cells in the organ of Corti is defined by the cell-cycle inhibitor, p27^kip1^ [22]. Similarly, bone morphogenetic protein 4 (BMP4) marks the incipient posterior crista by E11.5, followed shortly thereafter by *BMP4* delineation of the anterior and lateral cristae [23]. By E13, lunatic fringe expression is detectable in the presumptive utricular macula and saccular macula [23]. Thus, E11.5 is an appropriate developmental time point to interrogate molecularly-defined otic progenitors that will contribute to the auditory and vestibular sensory patches. The second reason E11.5 progenitors were targeted is that their infection with lineage virus is predicted to label multicellular clones of substantial cellular diversity. Tritiated thymidine birth-dating studies indicate that 50–70% of otic precursors giving rise to inner and outer hair cells and spiral ganglion cells exit the cell cycle by E13.5 and that terminal mitoses are complete by E15.5 [24]. Provided that retroviral infection, reverse-transcription, and chromosomal integration of provirus occur within 24hr of otocyst injection and that the average cell cycle time of otic precursors is on the order of 12–18 hr, 0–4 mitotic divisions of labeled precursors are expected before the cells of the sensory organs pull out of division. Lastly, the opaque, maternally-derived decidual tissue that encases the organogenesis-stage mouse embryo has thinned sufficiently by E11.5 to permit transuterine microinjection of lineage virus by transillumination with a fiber optic light source [16], [17].

Thirty five sequence-validated, multi-cell clones were identified in the P6 mouse inner ear that confirmed lineage relationships among hair cells, supporting cells, and nonsensory epithelial cells in the posterior crista; between outer hair cells and a supporting cell type in the organ of Corti; and between auditory neurons and interdental cells of the spiral limbus. In addition, multi-cell clones limited to single cell types (auditory neurons, vestibular neurons, marginal cells, or intermediate cells) were observed. These data extend lineage studies to the developing mammalian inner ear and reveal some basic similarities with previous data obtained in the chicken embryo.

## Materials and Methods

### Production of the BOLAP viral library

**Figure 1:**
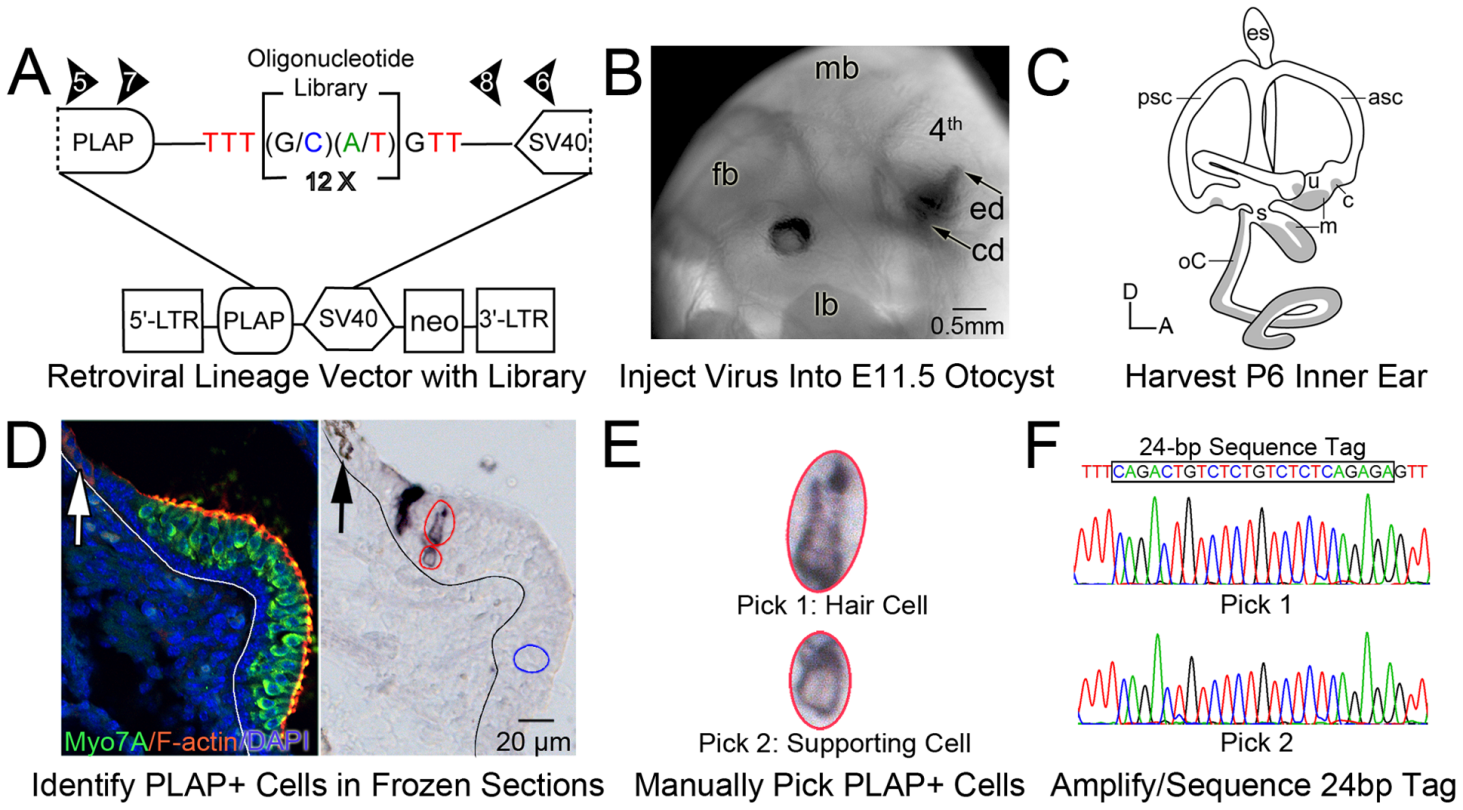
Retrovirus-mediated lineage analysis of the E11.5 mouse inner ear. A, schematic of the proviral form of the replication defective BOLAP murine retrovirus. PLAP is the genetically encoded lineage label. The 24-bp oligonucleotide library consists of approximately10^7^ unique 24-bp sequence tags. Abbreviations: LTR, long terminal repeat; neo, TnS neomycin resistance gene; SV40, simian virus 40 polyadenylation signal. B, lateral view of an E11.5 mouse embryo whose left otic vesicle was filled by transuterine microinjection with dye-tinged retroviral inoculum. The endolymphatic duct (ed) and cochlear duct (cd) are evident. Abbreviations: 4^th^, nascent 4^th^ ventricle of the hindbrain; fb, forebrain; lb, limb bud; mb, midbrain. C, schematic of the P6 mouse inner ear showing the approximate locations of the sensory organs in gray. Abbreviations: A, anterior; asc, anterior semicircular canal; c, crista ampularis; D, dorsal; es, endolymphatic sac; m, maculae; oC, organ of Corti; psc, posterior semicircular canal; s, saccule; u, utricle. D–F, analysis of a clone. D (left), a confocal image of a control P6 posterior crista demonstrating sensory hair cells (myosin7a [Myo7A], green), filamentous actin (F-actin; phalloidin, red), and cell nuclei (DAPI, blue). The white line demarcates the boundary between the sensory epithelium and the underlying mesenchyme (left). The white arrow indicates the position of the neural crest-derived melanin-expressing cells most proximal to the sensory epithelium of the crista (bright field image not shown). D, (right), PLAP-positive cells detected in a P6 posterior crista. The black line demarcates the sensory/nonsensory boundary. The black arrow indicates the position of the neural crest-derived melanin-expressing cells most proximal to the sensory epithelium. The larger purple cell (large red oval) is in the hair cell region. The small purple cell (small red oval) is in the supporting cell layer. The blue oval is a negative control pick. E, enlarged images of the hair cell and supporting cell outlined in D (right). F, sequence of the 24-bp oligonucleotide tags amplified from the hair cell (Pick 1) and supporting cell (Pick 2). The hair cell and supporting cell in the posterior crista share the same sequence tag suggesting they originated from a common progenitor and are lineally related.

BOLAP is a murine retroviral vector with an oligonucleotide library, schematically represented in Fig. 1A. The principle was to ligate a population of double stranded DNA molecules that includes a short degenerate region, [(G or C)(A or T)] repeated 12 times, into a retroviral vector DNA backbone. The construction of BOLAP was described previously [18].

The BOLAP retrovirus was produced in 293T cells [18], [25], [26]. Eight 10-cm plates of 293T cells were prepared at 40–50% confluency on Day 0. On Day 1, at 70% confluency, each plate was transfected with a pool of plasmids containing 6 µg BOLAP, 3 µg pMD gagpol plasmid [27], and 1 µg VSV-G envelope plasmid (pCL VSV-G, gift from Richard Mulligan, Harvard Medical School). Media was changed the following day and replaced with 5 mL of fresh media. On Days 3 and 4, the supernatant was collected and frozen. Supernatants were thawed and viruses concentrated by ultracentrifugation [26]. Viruses were titered by serial dilution onto 293T cells, as previously described [26]. Titers ranged from 7×10^6^ to 5×10^7^ infectious units/ml (iu/ml).

### Animal Use Ethics Statement

All procedures on mice were conducted to minimize pain and discomfort in strict accordance with the recommendations in the Guide for the Care and Use of Laboratory Animals of the National Institutes of Health. The animal care protocol was approved by the Oregon Health & Science University's Institutional Animal Care and Use Committee (OHSU Protocol Number: IS1286). Mouse survival surgeries were performed under sodium pentobarbital anesthesia [28] and post-procedural pain management included use of the non-steroidal anti-inflammatory meloxicam [29]. In an effort to meet ARRIVE Guidelines for reporting *in vivo* experiments [30], additional mouse procedural details are provided below and detailed in a recent publication [16].

### Timed pregnant breeding and mouse survival surgery

C57BL/6 males were crossed to ICR female mice to generate embryos for lineage analysis. Dams at E11.5 (12 noon on the day a vaginal plug was detected was considered embryonic day 0.5) were anesthetized with 60–65 µg/gram body weight of sodium pentobarbital solution containing 20.8 mg/mL MgSO_4_, 10% ethanol, and 40% propylene glycol [31]. Survival surgeries were conducted in a certified Envirco laminar flow clean bench that provides a class 100 sterile environment [16]. The uterine horns were externalized by ventral laparotomy and transilluminated with low intensity halogen light from a 7 mm diameter fiber optic guide. The uterine horn was gently palpated to reorient the embryo for transuterine microinjection of viral inoculum into the otic vesicle or fluorescent dextran into the hindbrain. Injection techniques are described in detail below. After transuterine microinjection, the abdominal wall and skin were closed with a running stitch using 6–0 (0.7 metric) Polysorb braided lactomer 9–1, CV-11, taper (GL-889, Syneture, United States Surgical). Prophylactic analgesic was achieved by subcutaneous injection of the nonsteroidal anti-inflammatory, Meloxicam (5 mg/kg body weight). Recovering dams were co-housed postoperatively and separated 1–2 days before parturition.

### Microinjection pipette fabrication

Thick-walled borosilicate glass capillary pipettes with an internal glass filament (BC150F-10; Harvard Apparatus) were pulled with a Flaming/Brown Model P-97 horizontal pipette puller (Sutter Instrument Co., Novato, CA) equipped with a 3 mm×3 mm box filament using the following program: pressure  = 200; heat  =  ramp value plus 3 units; pull  = 0; velocity  = 46; and time  = 110. The pulled pipettes were manually broken with forceps to 14–16 µm outer diameter under a Leica MZFLIII or MZ10F stereofluorescence microscope equipped with an ocular micrometer. Manually broken pipettes were beveled in 18.2 MΩ·cm water to 20 degrees with a K. T. Brown Type Beveler using the 104C abrasive plate (Sutter Instrument Co.) [31].

### Transuterine microinjection into the otocyst

The contrast agent fast green (Sigma-Aldrich) was added, in crystalline form, to 10 µL of concentrated viral inoculum and gently triturated 25 times with care taken to avoid any bubble formation. Insoluble particulates were precipitated with a 10 sec spin at 10,000 g in a microcentrifuge. Freshly beveled pipettes were backfilled with 10 µL of fast green/inoculum solution. The remaining inoculum was stored on ice until use. The loaded injection pipette was secured in a gasketed pipette holder compatible with the PicoSpritzer III microinjector (Parker Hannifin Corporation, General Valve Operation, Fairfield, NJ). Compressed nitrogen (>99% purity) was used to inject inoculum. The E11.5 otocysts were injected by sequential Picospritzer III pulses (15psi and 15 msec duration) until the entire lumen of the otocyst was filled as determined by visualization of the nascent endolymphatic duct dorsally and cochlear duct ventrally (Fig. 1B). Approximately 200–250 nL of inoculum was injected into an otocyst and one otocyst was injected per embryo.

### Transuterine microinjection into the hindbrain

Because not all embryos were accessible, those embryos whose otocysts were injected with the lineage virus received an additional injection of ∼250 nL of Alexa Fluor 488 conjugated dextran (10 mg/mL, aqueous; Invitrogen) into the nascent fourth ventricle of the hindbrain to enable postnatal selection of pups with virally-transduced inner ears [16]. The injections were conducted under mixed bright field/epifluorescence illumination using a Leica MZFLIII or MZ10F stereofluorescence dissecting microscope outfitted with an EGFP filter set and SOLA light engine (Lumencor, Beaverton, OR). At postnatal day 0 (P0), the hindbrain region of living pups was screened by epifluorescence stereomicroscopy to detect Alexa Fluor 488 fluorescence. Pups with green hindbrain fluorescence were identified by unique tattoos and returned to the dam. Non-fluorescent siblings were not tattooed and were used as controls for tissue preparation and alkaline phosphatase staining.

### Inner ear harvest and tissue processing

P6 pups were anesthetized by intraperitoneal injection of 60–65 µg/gram body weight of sodium pentobarbital anesthetic solution and decapitated. The head was fixed in 4% paraformaldehyde in calcium-free phosphate buffered saline (PBS; 137 m*M* NaCl, 2.7 m*M* KCl, 9.9 m*M* Na_2_HPO_4_, 2 m*M* KH_2_PO_4_, pH 7.2) at 4°C overnight with gentle agitation. The skin was removed and the skull was hemisected at the midline with a razor blade. The brain was removed from each hemisphere and cranial tissue was excised to isolate the temporal bone containing the inner ear and the associated ganglia. Individual inner ears were cryoprotected stepwise in 10%, 20%, and 30% sucrose in calcium-free PBS. Inner ears were infiltrated with OCT medium, reoriented inside cryostat tissue molds, and snap frozen on an aluminum plate equilibrated with liquid nitrogen. Frozen, embedded inner ears were stored at −80°C in tin foil pouches sealed inside plastic airtight sample bags.

### Tissue sectioning and lineage label detection

Inner ears were serially sectioned at 12–14 µm with a Microm HM 505E cryostat outfitted with a disposable Accu-Edge Low Profile microtome blade (Sakura FineTek USA, Inc). Individual tissue sections were transferred to Superfrost Plus microscope slides (Fisher Scientific), air dried for 30 min at 37°C and stored at −20°C. Slides containing sections from the same inner ear were thawed for 30 min at 37°C. Tissue sections were rehydrated in PBS, fixed in 4% paraformaldehyde in PBS (pH 7.2), and washed with three changes of PBS. PLAP was detected in cells with 1-Step NBT/BCIP Plus Suppressor solution (Thermo Scientific). The suppressor in the NBT/BCIP substrate solution was 1 mM levamisole, an inhibitor of endogenous AP activity that has no appreciable effect on PLAP activity. The progress of the PLAP reaction was monitored on uncoverslipped slides at 40× magnification with an Olympus SZ dissecting microscope with a long working distance (LWD) objective. When the signal from PLAP-positive cells was distinct from background, the reaction was terminated by washing the slide in three changes of PBS (pH 7.2). Developed slides were mounted in 90% glycerol-PBS (0.22 µm filtered) and coverslipped.

### Photomicroscopy

PLAP-positive cells were visualized by differential interference contrast microscopy using 10X/0.3NA, 20X/0.5 NA, and 40X/0.75NA objectives on a Leica DM 2500 epifluorescence microscope. Images of putative clones were obtained with a Leica DFC 420C cooled color camera driven by the Leica Application Suite Version 3.8.0.

### Picking PLAP-positive cells

Coverslips were removed from imaged slides by soaking in PBS and sections were washed in three changes of PBS to remove glycerol mounting medium. Excess PBS was removed from the slide and a drop of sterile MilliQ water was added to a tissue section containing PLAP-positive cells. A sterile, 30G (½ inch) Becton Dickinson Precision Glide needle mounted to a 1 mL sterile, disposable syringe was used for microdissection under a Leica MZFLIII dissecting scope with 1.0X LWD objective. The PLAP-positive cell and a small annulus of surrounding cells were mechanically freed from the section using the tip of the needle (Fig. 1D, E). The PLAP-negative tissue was separated from the PLAP-positive cell at the air/water interface of the water droplet, leaving behind the PLAP-positive “pick”. Separate PLAP-negative picks in close proximity to PLAP-positive cells were obtained in similar fashion and served as negative controls for PCR reactions. The location of each PLAP-positive (experimental) and PLAP-negative (control) cell pick was marked on the digital microscopy image of the putative clone. A new, 30G needle was used to transfer the PLAP-positive or PLAP-negative picks to separate PCR tubes containing 10 µL of proteinase K digestion buffer (50 m*M* KCl, 10 m*M* Tris-HCl [pH 7.5], 2.5 m*M* MgCl_2_, and 0.02% Tween 20) and 200 µg/mL proteinase K (Ambion). Picked cells were digested in their PCR tubes with a mineral oil overlay in a BioRad MyCycler by heating at 60°C for 2 hr. Proteinase K was inactivated by heating at 85°C for 20 min and 95°C for 10 min.

### Amplification of the 24 base pair oligonucleotide tag

The genomically incorporated 24-bp oligonucleotide sequence tag was amplified from picked cells by nested PCR. PCR reactions were assembled inside a laminar flow Airclean 600 PCR workstation (Air Clean Systems) dedicated *exclusively* to lineage PCR analyses. A 15 min UV irradiation sequence was initiated prior to all reaction assemblies to render contaminating DNA in the work area unsuitable for amplification. The first PCR (PCR1) was conducted with the flanking primers BOLAP 5 (5′-CCAGGGACTGCAGGTTGTGCCCTGT-3′) and

BOLAP 6 (5′-AGACACACATTCCACAGGGTCGAAG-3′). PCR2 was conducted with the internal primers BOLAP 7 (5′-GGCTGCCTGCACCCCAGGAAAGGAG-3′) and BOLAP 8 (5′-GGTCTCGGAAGCCCTCAGCCCAGTC-3′) (Fig. 1A). In practice, 40 µL of PCR buffer containing 10 µ*M* each of BOLAP 5 and BOLAP6 and 1 unit of High Fidelity Platinum Taq Polymerase (Invitrogen) was added to the 10 µL heat inactivated proteinase K digest. Cycling conditions for PCR1 were: denaturation at 93°C for 2.5 min; 33 cycles at 94°C for 45 sec, 63°C for 2min, and 72°C for 2 min; and polishing at 72°C for 5 min. PCR2 was performed by adding 3 µL of PCR1 to 47 µL of PCR buffer containing 10 µ*M* each of BOLAP 7 and BOLAP 8 and 1 unit of Taq polymerase. Cycling conditions for PCR 2 were: denaturation at 93°C for 2.5 min; 30 cycles at 94°C for 45 sec, 72°C for 2 min; and polishing at 72°C for 5 min.

### Sequencing the 24 base pair oligonucleotide tag

PCR2 reactions were resolved in a 1.5% Tris-acetate EDTA agarose gel to verify the expected 160 bp amplification product. PCR2 amplification products contained the 24-bp oligonucleotide tag and were sequenced in the Vollum Institute Automated DNA Sequencing Core using the BOLAP 7 primer and an ABI 3130XL sequencer (Fig. 1F).

### Assignment of clonal relationships

The cell type identity of PLAP-positive cells was ascertained histologically based on cell location, size, and morphology (representative example shown in Fig. 1 D, E). PLAP-positive cells that shared the same genomically-incorporated, 24-bp oligonucleotide tag sequence were assumed to originate from a common progenitor and to be members of the same clone (Fig. 1F). We detected 230 unique sequence tags from six inner ears. We did not detect the same sequence tag in cells from diverse anatomical locations in the same inner ear or in cells from different inner ears. These observations are consistent with the highly complex nature of the oligonucleotide library encoded in BOLAP.

## Results and Discussion

### Study focus and methodological overview

The focus of this study was on otocyst-staged precursors that give rise to differentiated cells in the auditory and vestibular sensory epithelia and neurons in the auditory and vestibular ganglia. The lineage vector incorporated a 24-bp oliogonucleotide library possessing a complexity of approximately 10^7^ unique sequence tags (Fig. 1A) [18]. The retrovirus was injected into the E11.5 mouse otic vesicle by transuterine microinjection (Fig. 1B) and embryos were carried to term and born naturally [16], [17]. PLAP was detected in cryostat tissue sections of the P6 inner ear using a chromogenic substrate (Fig. 1C, D, right) [32]. Typically, a single retroviral particle infects a progenitor and the PLAP gene and unique sequence tag are asymmetrically integrated into one of the daughters during the first mitotic division post-infection [33], [34], [35]. Daughter cells subsequently share expression of PLAP and the same genomically integrated tag. Differentiated PLAP-labeled cells were mechanically picked from sections of the differentiated, postnatal inner ear and their oligonucleotide tags were amplified, sequenced, and compared (Fig. 1E, F) [32], [36].

### Optimizing BOLAP infection for lineage analysis of the E11.5 mouse inner ear

To ascertain infection dynamics of the BOLAP retroviral vector in the developing mouse inner ear, 20 otic vesicles in 20 E11.5 embryos from two dams were injected with viral inoculum at 5×10^7^ iu/mL and harvested at E18.5. Serial cryostat sections of the injected inner ears from this cohort demonstrated PLAP-positive cells distributed throughout the inner ear with dozens of cells clustered closely together (data not shown). The large number of PLAP-positive cells in close apposition confounded efforts to manually pick cells for clonal analysis.

To optimize BOLAP infection for reliable and efficient cell picks, the concentrated inoculum was diluted 5 fold to 1×10^7^ iu/mL and injected into 38 otic vesicles in 38 E11.5 embryos from six dams. Injected embryos and uninjected control littermates were born naturally and harvested at P6 when the inner ear is morphologically mature and constituent cell types are spatially distributed similar to the adult inner ear (Fig. 1C). Thirty two of the 38 inner ears were not analyzed because there were numerous PLAP+ cells tightly clustered preventing interrogation of complex lineages (12/32), or the inner ears possessed no PLAP+ cells in anatomical regions of interest (20/32). Six of the 38 inner ears from this cohort were selected for subsequent clonal analysis because they contained PLAP-positive cells in sensory, nonsensory, and neuronal regions of the inner ear (Table 1). The six ears contained PLAP-positive cells in the sensory epithelium of the posterior crista and 5/6 ears contained PLAP-positive cells in the sensory epithelium of the organ of Corti (Table 1). Five of six inner ears presented PLAP-positive cells in the nonsensory epithelium of the posterior crista, the spiral limbus, and the auditory ganglion and nerve. PLAP-positive cells were least frequently detected in the sensory and nonsensory epithelia of the anterior and lateral cristae and the utricular and saccular maculae (Table 1).

**Table 1:** Qualitative Distribution of PLAP+ Cells in Sensory, Nonsensory, and Neuronal Regions of the P6 Mouse Inner Ear Injected with BOLAP Retroviral Lineage Vector at E11.5.

Region	Location§	P6 Inner Ear Specimen Identityδ					
		HJ- 5	HJ- 6	HJ- 8	HJ- 9	HJ- 11	HJ- 12
Sensory	Posterior crista	√	√	√	√	√	√
	Organ of Cortiψ	√	√	√		√	√
	Anterior crista	√					
	Lateral crista		√				
	Utricular macula		√				
	Saccular macula	√					
Nonsensory	Posterior crista	√	√		√	√	√
	Spiral limbus	√	√	√		√	√
	Stria vascularis	√	√		√		√
	Reissner's membrane	√	√		√		√
	Spiral ligament	√	√	√			
	Saccule	√			√		
	Anterior crista	√					√
	Utricle		√				√
	Lateral crista		√				
Neuronal	Auditory ganglion and nerve	√	√	√		√	√
	Vestibular ganglion and nerve	√	√		√		√

§ defined by microscopic evaluation of serial 14 µm cryostat sections.

δ two or more PLAP+ cells in a location earned a positive score (checkmark). HJ are author initials followed by the unique specimen identification code.

ψ PLAP+-cells were most frequently detected in the base and middle turn of the cochlea and rarely in the apex.

### Cell picks and false positive rate

In the six inner ears analyzed, 655 cell picks were made: 547 (83.5%) were PLAP-positive cell picks and 108 (16.5%) were PLAP-negative cell picks as defined in the Methods section (Table 2). PLAP-negative picks served as negative controls for nested PCR amplification of the degenerate 24-bp sequence tag (Fig. 1A, F). The detection of a PCR product in 3 PLAP-negative picks established a false positive rate of 2.8% (Table 2) and is similar to the 5% false-positive rate defined for picks of chicken inner ear tissues infected with the CHAPOL (chick alkaline phosphatase with oligonucleotide library) retroviral lineage vector [12]. All 3 PLAP-negative picks that generated a PCR product were made in the spiral ganglion where non-neural cells with variable PLAP expression were found. The low false positive rate is therefore unlikely to reflect gross contamination of the nested PCR reactions. Rather, it may result from inadvertent inclusion of a weakly expressing PLAP-positive cell in the negative pick or from inclusion of an infected cell whose PLAP expression was silenced.

**Table 2:** Quantification of Cell Picks and Sequence Tags from the P6 Mouse Inner Ear Injected with BOLAP Retroviral Lineage Vector at E11.5.

Description		Quantity	Percentage
Number of PLAP+ and PLAP− cell picks		655	na§
	PLAP+ cell picks	547	83.5
	PLAP− cell picks	108	16.5
Number of PLAP- cell picks that returned a sequence tag		3	2.8
Number of PLAP+ cell picks that returned a single sequence tag or multiple tags		285	51.1
	Single sequence tags	230	80.7
	Multiple sequence tags	55	19.3δ
Distribution of Single and Multicellular Clones:			
	Single cell clonesψ	195	84.8
	Clones with 2 or more cells	35	15.2

§ not applicable.

δ the majority of the multiple sequence tags were detected in picks of the spiral ganglion.

ψ PLAP+ cell picks whose sequence tag did not match with any other sequence tag.

### Multiple sequence tag detection rate

In the six inner ears analyzed, 285 picks of PLAP-positive cells (51.1%) returned a PCR product after nested amplification and 55 of these (19.3%) contained multiple sequence tags precluding assignment of clonal relationships (Table 2). Detection of multiple sequence tags could result from the inclusion of two or more infected cells from different lineages in the tissue pick; co-infection of a precursor with one or more viral particles that carry different sequence tags [37]; or silent infection where a cell infected with BOLAP is sufficiently transcriptionally inactive so as not to produce enough PLAP to be histochemically detectable [38]. In the case of the mouse inner ear, the vast majority of the multiple sequence tags were detected in picks from the spiral ganglion where small, neural crest-derived Schwann cells with heterogeneous PLAP expression likely contaminate the picks and contribute the errant sequence tag(s).

### Single cell clone detection rate

A single sequence tag was identified in 230 PLAP-positive picks. These tagged cells could therefore be compared to cells within the same inner ear to infer lineal relationships. However, the vast majority (84.8%) of the single-sequence tags identified in labeled cells across a given inner ear were unrelated to one another, therefore preventing inference of lineage relationships among the tagged cells. The high single cell clone detection rate could be accounted for by too infrequent picks of small, spatially dispersed clones, a possibility minimized in these experiments by tuning the BOLAP infection rate and densely picking a large number of PLAP-positive cells in each serial tissue section (Table 2). Another explanation is that tissue sections containing lineally related cells were lost during cryostat sectioning, a possibility effectively eliminated by a “lost section” rate of <1%. An alternative explanation for the high single cell clone detection rate is viral integration during the M-phase preceding the final precursor cell division (Table 2). An extended temporal requirement for retroviral infection or integration into otic precursors, or a cell cycle time of >18 hr, could account for integration at or after E13.5 when the majority of precursors have engaged their terminal mitosis [24]. Auditory ganglion neurons were the most frequently identified single cell clone type followed by cells in the stria vascularis and Reissner's membrane (Table 3). Single cell clones were also found in the posterior crista, spiral limbus, and maculae (Table 3).

**Table 3:** The Identity of Sequence Tag-Validated Single Cell Clones in the P6 Mouse Inner Ear after Injection with BOLAP Retroviral Lineage Vector into the E11.5 Mouse Otic Vesicle.

Region		Quantity (Total: 195 clones)
Posterior Crista		
	Hair cell	18
	Nonsensory epithelial cells	15
Organ of Corti		
	Hair cell	10§
	Supporting cell	16δ
	Claudius' cell	13ψ
Auditory ganglion neuron		53
Spiral limbus		
	Interdental cell	10
	Mesenchyme	10
Stria Vascularis		25
Reissner's membrane		15
Saccular macula		
	Hair cell	3
	Supporting cell	1
	Nonsensory epithelial cell	2
Utricular macula		
	Hair cell	2
	Supporting cell	1
	Nonsensory epithelial cell	1

§ 7 in base; 3 in middle turn.

δ 7 in base; 9 in middle turn.

ψ 10 in base; 3 in middle turn.

A previous retroviral lineage analysis of the nascent chick otocyst demonstrated that 40% of clones were of the single cell type [14]. Rather than indicating a difference between species, the lower rate of single cell clone detection in the chicken inner ear likely resulted from injection into the otocyst soon after its vesicularization, which is ∼24 hr earlier that the E11.5 mouse otocyst injections conducted here. The earlier injection deployed in the chick resulted in integration of the retroviral construct at an earlier developmental stage than in the mouse, permitting the observation of additional mitotic events and producing a higher frequency of multicellular clones. These data support the prediction that retroviral lineage analysis conducted at earlier time points than E11.5 will lead to larger clones with more complex cellular composition. To achieve nascent otocyst injections in the mouse, an accessory imaging modality such as ultrasound will be required to guide transuterine microinjection through the opaque extraembryonic tissues [39], [40].

### Multicellular clone detection rate

Thirty-five of the 230 (35/230, 15.2%) single sequence tags defined clones with two or more cells (Table 2). The multicellular clones consisted of an average of 3.4 PLAP-positive cells (range: 2–18 cells; Table 4). The clones are grouped in Table 4 by anatomical location to facilitate comparisons between clones in a defined region of the inner ear. An estimate of the dispersion of cells in a clone is provided by the clonal spread value, the maximum distance between two cells of a clone measured perpendicular to the plane of section. A clone contained within the same 14 µm tissue section is nondispersed and two related cells three sections apart are spread by a maximum of 42 µm. A description of several representative, sequence-validated multicellular clones follows.

**Table 4:** The Anatomical Location, Cellular Composition, and Dispersion of Thirty Five Sequence Tag-Validated Multicellular Clones in the P6 Mouse Inner Ear after Microinjection of the BOLAP Retroviral Lineage Vector into the E11.5 Mouse Otic Vesicle.

Inner Ear Specimen Identity	Clone Number (Figure Number)	Anatomical Location	Number of Cells in the Clone	Cellular Composition†	Clonal Spread§ (µm)
HJ-9	25 (1 and 2)	Posterior crista	4	Hair cells	84
			8	Supporting cells	
			6	Indeterminate	
HJ-6	19 (3)	Posterior crista	2	Hair cells	42
			3	Nonsensory epithelial cells	
HJ-6	16	Posterior crista	2	Hair cells	
			3	Nonsensory epithelial cells	56
HJ-5	08	Posterior crista	3	Nonsensory epithelial cells	(–)δ
HJ-12	31	Posterior crista	3	Hair cells	56
HJ-5	05 (4)	Organ of Corti	1	Outer hair cell	(–)
			1	Supporting cell	
HJ-5	01	Organ of Corti	2	Hair cells	(–)
HJ-5	04	Organ of Corti	1	Hair cell	70
			1	Supporting cell	
HJ-5	06	Organ of Corti	4	Claudius' cells	406
HJ-5	03	Auditory ganglion	2	Neurons	378
HJ-5	07	Auditory ganglion	3	Neurons	238
HJ-5	09	Auditory ganglion	4	Neurons	140
HJ-5	10	Auditory ganglion	6	Neurons	224
HJ-5	11	Auditory ganglion	3	Neurons	56
HJ-6	12	Auditory ganglion	2	Neurons	112
HJ-6	13	Auditory ganglion	2	Neurons	(–)
HJ-6	15	Auditory ganglion	3	Neurons	42
HJ-6	21	Auditory ganglion	2	Neurons	(–)
HJ-8	22	Auditory ganglion	2	Neurons	(–)
HJ-11	26	Auditory ganglion	2	Neurons	(–)
HJ-11	27	Auditory ganglion	4	Neurons	70
HJ-12	33	Auditory ganglion	2	Neurons	(–)
HJ-12	32 (5)	Auditory ganglion	3	Neurons	28
		Spiral limbus	1	Interdental cell	
HJ-9	24	Vestibular ganglion	4	Neurons	140
HJ-12	29	Auditory ganglion	1	Non-neuronal cell	224
		Spiral limbus	3	Mesenchymal cells	
HJ-12	30	Auditory ganglion	1	Non-neuronal cell	210
		Spiral limbus	2	Mesenchymal cells	
HJ-5	02	Spiral limbus	4	Interdental cells	140
HJ-12	28	Spiral limbus	2	Interdental cells	140
HJ-8	23	Spiral limbus	2	Non-epithelial cells	(–)
HJ-6	14	Scala vestibuli/media	2	Reissner's membrane cells	28
HJ-6	20	Scala vestibuli/media	2	Reissner's membrane cells	(–)
HJ-6	18	Scala vestibuli/media	2	Reissner's membrane cells	42
HJ-12	34	Scala vestibuli/media	4	Reissner's membrane cells	84
		Stria vascularis	1	Basal cell	
		Stria vascularis	1	Undefined cell typeψ	
HJ-6	17	Stria vascularis		Marginal cells	70
HJ-12	35	Stria vascularis		Intermediate cells	308

† anatomical and morphological criteria were used to determine cell type identity.

§ the maximum distance between two cells of a clone measured perpendicular to the plane of section.

δ cells in the clone were constrained to the same tissue section and are nondispersed.

ψ PLAP+ cell in the basal cell layer was sectioned by the cryostat blade confounding morphological analysis.

### Vestibular sensory hair cells and their supporting cells are clonally related

**Figure 2:**
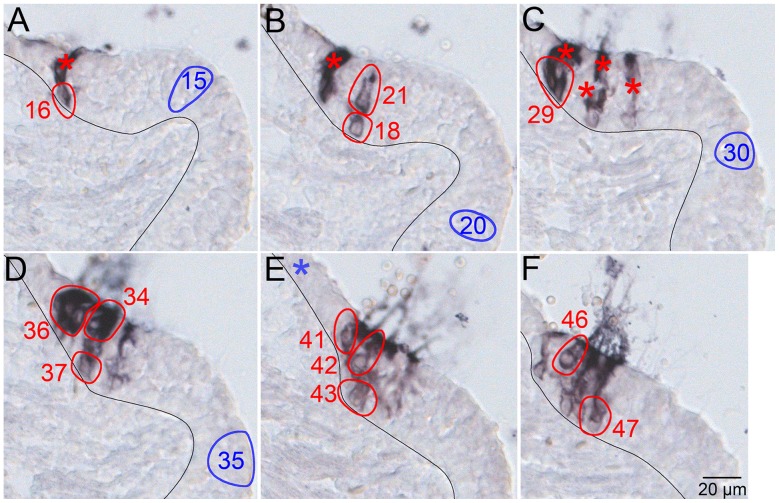
Sensory hair cells and supporting cells in the posterior crista are clonally related. A–F, serial, 14 µm sections of a P6 posterior crista demonstrating the PLAP-positive constituents of a sensory clone. The black line demarcates the boundary between the sensory epithelium (right) and the underlying mesenchyme (left) of the crista ampullaris. The red ovals are picks of PLAP-positive cells that returned the identical sequence tag (see Fig. 1) and define the clone. The blue ovals (15, 20, 30, and 35) are control picks of PLAP-negative tissue that did not generate a PCR product. The blue asterisk (Panel E) indicates there was an additional control pick in the nonsensory epithelium just beyond the cropped image. The red asterisks indicate the location of PLAP-positive cell picks that failed to return a PCR product.

Five multicellular clones were identified in four P6 posterior cristae, and four of these clones contained cells in the sensory epithelium (Table 4). The largest sensory clone (Clone 25) contained 18 cells in six serial sections: 4 vestibular hair cells, 8 supporting cells, and 6 cells of indeterminate identity at the tapered edge of the crista (Fig. 2). Hair cells (picks 21, 41, 42, and 46 in Fig. 2B, E, F) are situated above the supporting cell layer and present rounded basal regions that taper apically and terminate on the surface of the epithelium. Supporting cells (picks 16, 18, 29 [3 cells], 37, 43, and 47 in Fig. 2A–F) are basally located and have ovoid to rounded cell bodies. The density of the PLAP reaction product in picks 34 (2 cells) and 36 (4 cells) precludes unambiguous identification of individual cell types (Fig. 2D). The apical localization in the sensory epithelium suggests hair cell identity for cells in pick 34 (compare with the distribution of Myo7a-positive vestibular hair cells in Fig. 1D, left). Localization to the peripheral edge of the crista suggests nonsensory epithelial cell identity for cells in pick 36. Regardless, picks 34 and 36 returned the same sequence tag as the hair cells and supporting cells indicating at least one cell in each cluster is a constituent of the clone. These data indicate that vestibular hair cells and supporting cells are clonally related. In addition, the data raise the possibility that nonsensory epithelial cells bordering the crista may be related to the vestibular sensory epithelium.

**Figure 3:**
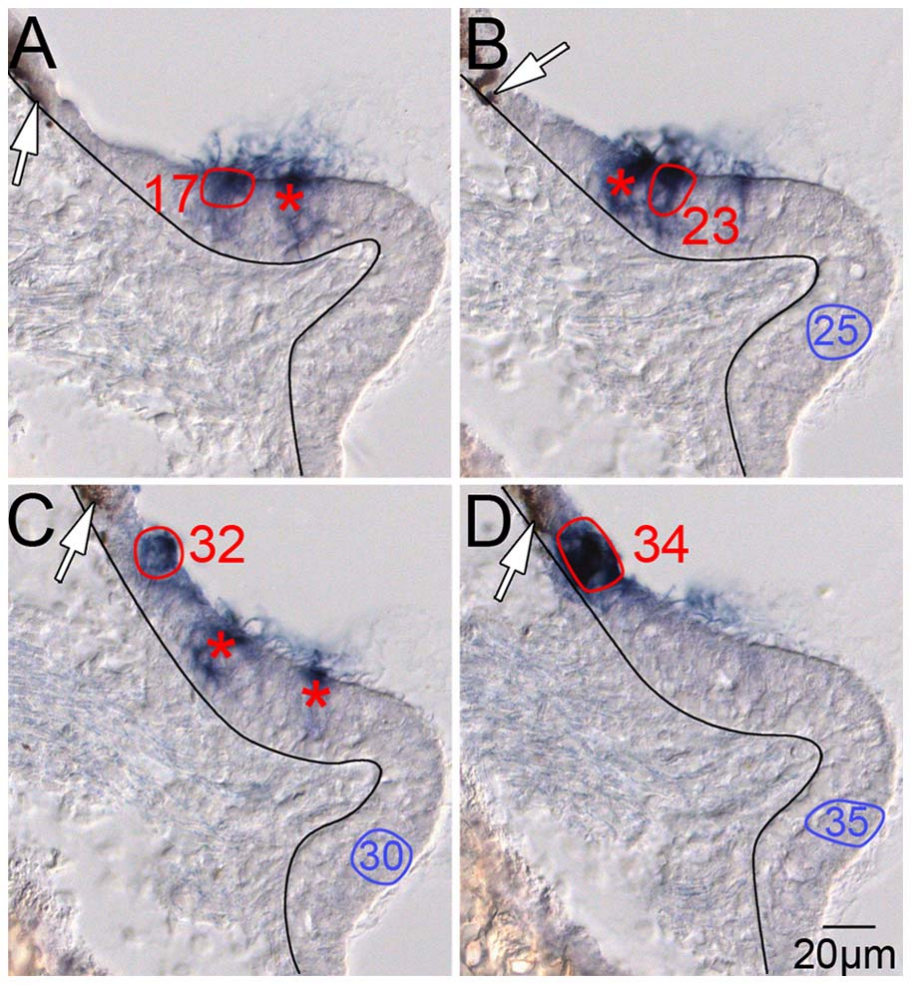
Sensory hair cells and nonsensory epithelial cells of the posterior crista are clonally related. A–D, serial, 14 µm sections of the posterior crista demonstrating the PLAP-positive constituents of a mixed sensory/nonsensory epithelial clone. The black line demarcates the boundary between the sensory epithelium (right) and the underlying mesenchyme (left) of the crista ampullaris. The red ovals are picks of PLAP-positive cells that returned the identical sequence tag. Picks 17 and 23 are in the hair cell layer of the crista while picks 32 and 34 are proximal to neural crest-derived melanocytes (white arrows) in the adjacent, nonsensory epithelium (see also Fig. 1, panel D, left, white arrow). The blue ovals (25, 30, and 35) are control picks of PLAP-negative tissue that did not generate a PCR product. The red asterisks indicate location of PLAP-positive cell picks that failed to return a PCR product.

To further address this issue, another clone (Clone 19) that spanned the sensory/nonsensory epithelial transition zone in the posterior crista was evaluated (Table 4). The location of melanocytes (Fig. 3A–D, white arrow) marks the edge of the nonsensory epithelium. A sensory/nonsensory epithelial transition zone extends from the melanocyte edge to the base of the elevated, thickened sensory epithelium of the crista ampullaris. Picks 32 and 34 (Fig. 3C, D, respectively) target PLAP-positive cells 20–30 µm from the melanocytes within the transition zone. Picks 17 and 23 (Fig. 3A, B, respectively) are in the hair cell layer of the crista >60 µm from the melanocytes. All four picks returned the same sequence tag. These data indicate that nonsensory epithelial cells and sensory hair cells in the posterior crista can be clonally related. Furthermore, we obtained additional confirmation of this lineage relationship from another nonsensory epithelial/hair cell clone from the same crista containing two hair cells and two nonsensory epithelial cells (Table 4, Clone 16).

### Outer hair cells and a supporting cell type are clonally related

**Figure 4:**
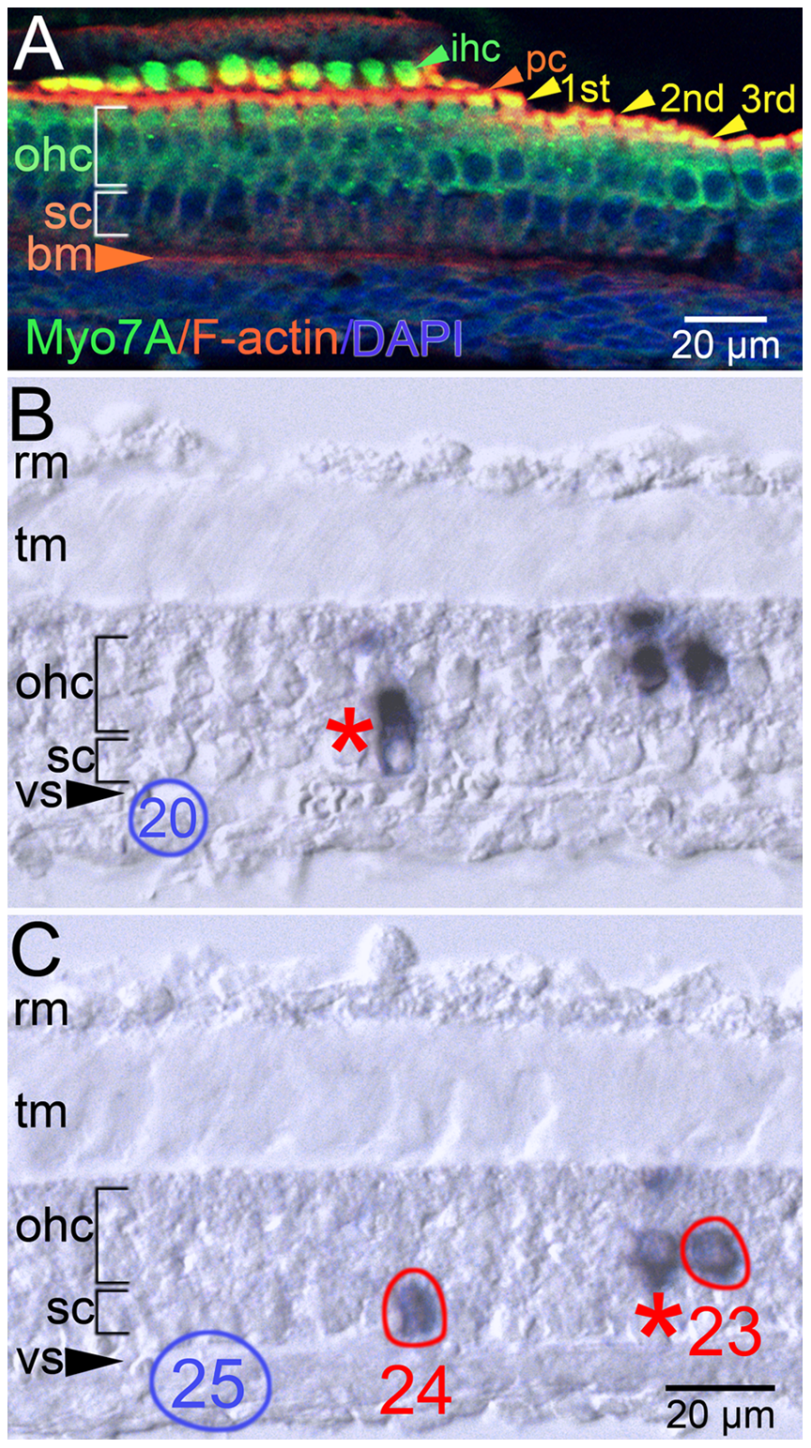
Outer hair cells and a supporting cell type are clonally related. A, confocal projection of an oblique, 14 µm section of a control P6 cochlear duct. Sensory hair cells (myosin7a [Myo7A], green), filamentous actin (F-actin, red), and cell nuclei (DAPI, blue) are detectable in this atypical histological presentation of the organ of Corti. The Myo7A-positive inner hair cells (ihc) are separated from the 1^st^, 2^nd^, and 3^rd^ rows of Myo7a-positive outer hair cells (ohc, wide bracket) by the F-actin-enriched apices of pillar cells (pc). The supporting cell (sc) nuclei occupy a region beneath the Myo7a-positive outer hair cells (ohc) indicated by the narrow bracket. B, C, oblique,14 µm, serial cryostat sections of a P6 organ of Corti in a similar orientation as in panel A presenting PLAP-positive cells of a clone. The supporting cell in pick 24 and the outer hair cell in pick 23 returned the identical sequence tag. The supporting cell in panel B (red asterisk) and the hair cell in panel C (red asterisk) did not return an oligonucleotide sequence. Negative control picks 20 (blue oval, panel B) and 25 (blue oval, panel C) did not generate a PCR product. The atypical plane of section precludes clear identification of the supporting cell type in the ohc clone. Abbreviations: bm, basilar membrane; rm, Reissner's membrane; tm, tectorial membrane; vs, vas spirale (tangential section).

Four multicellular clones in the cochlear duct of one P6 inner ear contained cells in the organ of Corti (Clones 1, 4, 5, and 6 in HJ-5, Table 4). All four clones were identified in tangential sections of the cochlear duct resulting in atypical histological presentations of the organ of Corti. To facilitate identification of cell types in these inner ears, a control P6 inner ear was oriented similarly, sectioned, and stained for the hair cell marker, Myosin 7a (Myo7a), filamentous actin (F-actin), and with a nuclear marker (DAPI; Fig. 4A). The plane of section presents an abneural to neural view with the apical portion of Myo7a-positive inner hair cells (ihc) and F-actin-positive pillar cells (pc) most distal in the image (Fig. 4A). Myo7a- and F-actin-positive stereociliary bundles from all three rows of outer hair cells (ohc) are evident (Fig. 4A). Most critically, the DAPI-positive outer hair cell nuclei are arrayed in distinct rows above the Myo7a-negative supporting cell layer establishing stratified outer hair cell and supporting cell layers (brackets, Fig. 4A). Clone 5 was detected in the middle turn of the cochlea in two serial, tangential sections of the cochlear duct (Fig. 4B, C). Three PLAP-positive cells were identified with cell bodies in a stratified organization occupying the presumptive outer hair cell or supporting cell layers (Fig. 4B, C). The registration of the three PLAP-positive cells between adjacent sections suggests that the three PLAP-positive cells were cut by the cryostat blade displacing parts of two outer hair cells (Fig. 4B, C, ohc layer, right) and one supporting cell (Fig. 4B, C, sc layer, left) into adjacent sections. The supporting cell in pick 24 and the outer hair cell in pick 23 (Fig. 4C) returned the same sequence tag and are lineally related. The precise identity of the supporting cell type is unclear, though the proximity of its nucleus to the vas spirale (vs; Fig. 4B, C) suggests either outer pillar cell or phalangeal cell identity.

Two additional sensory clones were detected in the organ of Corti in this specimen (HJ-5) consisting of two hair cells (Clone 1) and one hair cell and one supporting cell (Clone 4; Table 4). In both clones, efforts to unambiguously define hair cell and supporting cell types were confounded by an atypical presentation in the cryostat sections of the cochlear duct. The final organ of Corti clone consisted of four Claudius' cells spread over 406 µm (29 sections). In summary, outer hair cells can be related to a supporting cell type in the organ of Corti and multiple Claudius' cells can originate from a single common progenitor.

### Auditory and vestibular ganglion neurons are clonally related to cells of the same type

Fourteen multicellular clones were identified in the ganglia of the inner ear: 13 clones in the auditory ganglion (5/6 inner ears) and one clone in the vestibular ganglion (Table 4). Cells were identified as neurons by their soma size (6–9 µm diameter) and the presence of large circular-to-oval-shaped nuclei. The sole vestibular ganglion clone consisted of four neurons and twelve of thirteen auditory ganglion clones consisted of two to six neurons. While both shared and separate lineages give rise to acoustic and vestibular neurons in the chick inner ear [13], the paucity of vestibular ganglion clones labeled by E11.5 injections precluded sufficient interrogation of a putative shared lineage between the two ganglia in the mouse inner ear.

**Figure 5:**
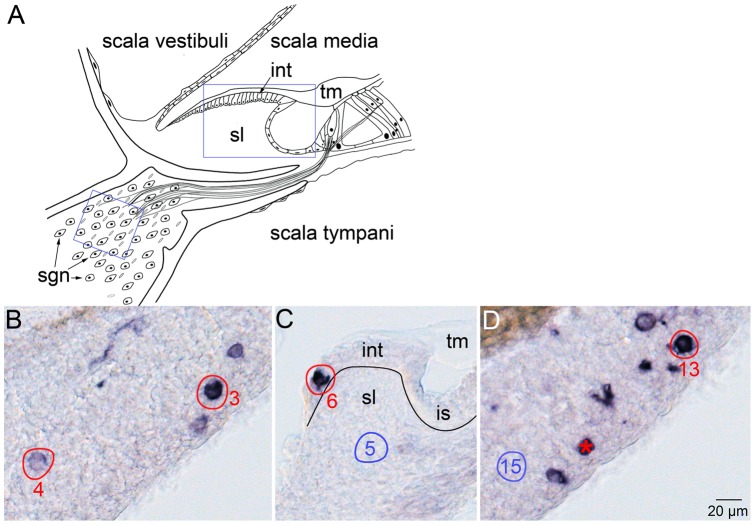
Spiral ganglion neurons and interdental cells are clonally related. The tectorial membrane (tm) overlies the interdental cells and the apical surface of the organ of Corti. Spiral ganglion neurons (sgn) occupy the auditory ganglion whose fibers innervate sensory hair cells. The large blue box represents the location of the section in panel C and the small blue box represents the location of the sections in panels B and D. Schematic modified from Davis 1962 [50]. B–D, serial, 14 µm sections with PLAP-positive cells in the spiral ganglion (panels B, D) and the epithelial lip of the spiral limbus (panel C). The PLAP-positive cells in picks 3, 4, and 13 have large (∼8 µm diameter) cell bodies located in the spiral ganglion consistent with neuronal morphology. The PLAP-positive cell in pick 6 is in the epithelial lip of the spiral limbus formed by interdental cells. The black line indicates the boundary between the epithelial and mesenchymal layers of the cochlear duct. The spiral ganglion neurons and the interdental cell share the same 24-bp sequence tag. Picks 5 and 15 are negative control picks and the asterisk indicates a spiral ganglion neuron that did not return a PCR product. Abbreviations: is, inner sulcus.

An auditory ganglion/nonsensory epithelial clone of particular interest (Clone 32, Table 4) consisted of three neurons (picks 3, 4, and 13; Fig. 5A, C) and an epithelial cell in the spiral limbus of the cochlea (pick 6, Fig. 5B). The localization of the cell in this nonsensory domain, its basal juxtaposition to the underlying mesenchyme, and its apical association with the overlying tectorial membrane are consistent with interdental cell identity. There is precedent for a clonal relationship between neuronal and nonsensory epithelial cells in the chick inner ear. Acoustic neurons in the chick inner ear are clonally related to nonsensory epithelial cells located between the utricular macula and the lateral crista in the utricle-lateral ampulla junction [13]. While additional clonal data will be required to definitively validate the auditory neuron/nonsensory epithelial relationship in the mouse inner ear, the data presented here suggest a shared lineage between auditory ganglion neurons and interdental cells of the spiral limbus.

### Clones in the spiral limbus, Reissner's membrane, and stria vascularis

Small clones containing 2–4 cells were identified in the spiral limbus (3/6 ears); Reissner's membrane (2/6 ears); or the stria vascularis (2/6 ears; Table 4). Interdental cells (Clones 2 and 28), marginal cells (Clone 17), and intermediate cells (Clone 35) were lineally related to cells of the same type (Table 4). Interdental and intermediate cell clones contained cells dispersed up to 140 and 308 µm, respectively. These data indicate substantial cellular displacement of clonally-related cells during morphogenesis of the spiral limbus and stria vascularis.

### Summary and Future Directions

The vast majority of labeled cells observed in this study were members of single cell clones (Table 2), suggesting that retroviral integration occurred during the terminal mitotic division. This is in keeping with the fact that the single copy of the viral genome of a gammaretrovirus cannot integrate into the host chromatin until after the nuclear envelope breaks down during M phase [35]. In effect, injection of lineage virus into the E11.5 otocyst primarily reported clonal relationships derived from E12.5 or older precursors. The lack of labeled cells in the anterior and lateral cristae, the maculae, and to a large extent the organ of Corti in this dataset indicate that progenitors giving rise to these sensory epithelia are not readily accessible through E11.5 otocyst injections of BOLAP. An alternative approach would be to assess whether Cre-Lox-based lineage technologies using advantageous Brainbow variants would be informative in the developing mouse inner ear [41], [42], [43].

Genetic fate mapping has previously shown that Neurogenin1-positive otic epithelial cells can differentiate as auditory or vestibular neurons, hair cells and supporting cells in the maculae, or non-sensory epithelial cells surrounding the maculae [44]. In the present study, auditory neurons in the spiral ganglion were the most frequent single cell clone type detected, and PLAP-positive cells were rarely seen in the maculae. How can the genetic fate mapping and retroviral lineage data be reconciled? Retroviral silencing of PLAP expression in sensory and nonsensory cells of the maculae could account for the failure to detect predicted clonal relationships between neurons and cells in the maculae. To address this possibility, we picked 20 PLAP-negative cells in the maculae of 3 inner ears with robust PLAP expression in spiral ganglion neurons and failed to detect a PCR2 product while 4 picks of PLAP-positive cells generated the expected PCR2 product. We conclude that retroviral silencing in the maculae, if present, is not widespread. We favor an alternative explanation whereby microinjected retroviral inoculum leaks out of the ventral otocyst where neuroblasts are delaminating [44], [45], [46]. The extra-otic inoculum would bathe delaminated neuroblasts and enhance the probability of their infection while the leakage of inoculum would reduce the number of viral particles within the otocyst capable of infecting the neurogenin-expressing precursors. Consistent with this interpretation is the frequent observation of PLAP-positive mesenchymal issue adjacent to the auditory ganglion that is also likely infected by leaked viral particles, a phenomenon that was also observed after retroviral injections into the chick otocyst [12]. The most compelling insights into cell fate determination in the inner ear will arise from the intersection of genetic fate maps with clonal analyses of mitotic progenitors.

Eight multicellular clones contained at least one PLAP-positive cell whose identity could not be definitively determined (Clones 4, 5, 14, 18, 20, 25, 29, and 34; Table 4). In seven of these clones, the constraining factor was the excessive deposition of PLAP reaction product in infected cells despite efforts to modulate the reaction in real time with microscopic monitoring. PLAP is anchored to the plasma membrane via hydrophobic residues in its C terminus permitting exquisite identification of cells based on complete labeling of their processes [47], [48]. Unfortunately, variable PLAP expression was often observed. Heterogeneous expression from the viral vector may reflect the fact that the viral genome integrates randomly into the genome and is subject to position-effect variegation and silencing [49]. In the case of the mouse inner ear, heterogeneous expression presented a confounding variable for unambiguous cell type identification in a significant number of multicellular clones. Recent lineage studies in the rodent retina using a retrovirus encoding membrane-anchored green fluorescent protein (mGFP) and an oligonucleotide library facilitated cell type identification by fluorescence imaging of mGFP-labeled cells [16]. In addition, immunostaining with cell-type- specific antisera to molecularly identify cell types is feasible in mGFP expressing cells, adding a layer of validation to the assignment of clonal constituents. While it is formally possible in our experiments to use an anti-PLAP antibody, the use of mGFP would make the approach far more efficient. The potential use of fluorescent activated cell sorting for isolation of clonal constituents and determination of cell type identity is also worthwhile to consider. Future lineage studies in the mouse inner ear will exploit ultrasound-guided transuterine microinjection of the GFP-encoding lineage vector to mark younger progenitors which should thus generate a larger number of multicellular clones to explore a greater range of lineage relationships throughout the mammalian inner ear.
